# USP54 is a potential therapeutic target in castration-resistant prostate cancer

**DOI:** 10.1186/s12894-024-01418-7

**Published:** 2024-02-06

**Authors:** Cheng Zhou, Xuehu Zhang, Hangbin Ma, Yinghao Zhou, Yibo Meng, Chenchen Chen, Guowei Shi, Wandong Yu, Jun Zhang

**Affiliations:** Department of Urology, Shanghai Fifth People’s Hospital, Fudan University, No. 801, Heqing Road, Minhang District, Shanghai, 200240 P.R. China

**Keywords:** USP54, Prostate cancer, Deubiquitinase, CRPC

## Abstract

**Background:**

USP54, a ubiquitin-specific protease in the deubiquitinase (DUB) family, facilitates the malignant progression of several types of cancer. However, the role of USP54 in prostate cancer (PCa), especially castration-resistant prostate cancer (CRPC), remains unknown.

**Methods:**

We established the CRPC LNCaP-AI cell line from the hormone-sensitive prostate cancer (HSPC) LNCaP cell line. RNA-Seq was utilized to explore DUB expression levels in LNCaP and LNCaP-AI. USP54 was knocked down, and its effects on cell growth were evaluated in vitro and in vivo. Bioinformatics analyses were conducted to explore signaling pathways affected by USP54 in PCa. Quantitative polymerase chain reaction was used to confirm key signaling pathways involved.

**Results:**

USP54 was the most strongly upregulated DUB in LNCaP-AI cells compared with LNCaP cells. USP54 levels were higher in PCa than in normal tissues. USP54 silencing suppressed the proliferation of PCa cell lines, both in vitro and in vivo. USP54 expression was positively correlated with the androgen receptor (AR) signaling level in PCa samples, and USP54 knockdown inhibited AR signaling in PCa cells.

**Conclusions:**

USP54 was upregulated during HSPC progression to CRPC. USP54 depletion suppressed CRPC cell proliferation both in vitro and in vivo. USP54 may facilitate PCa progression by regulating AR signaling.

**Supplementary Information:**

The online version contains supplementary material available at 10.1186/s12894-024-01418-7.

## Introduction

Prostate cancer (PCa) is a common type of carcinoma; millions of new cases are diagnosed worldwide every year [1, 2]. Most newly diagnosed PCas are dependent on androgens and thus termed hormone-sensitive prostate cancers (HSPCs). Patients with localized disease who receive androgen-deprivation treatment (ADT) generally experience initially satisfactory outcomes [3]. However, most patients subsequently develop castration-resistant prostate cancer (CRPC), which has more aggressive biology and shorter overall survival [4].

Ubiquitination is a form of post-translational modification that regulates protein function and initiates protein degradation [5]. Various deubiquitinases (DUBs), including ubiquitin-specific proteases, remove ubiquitin from substrates and thus eliminate ubiquitination signals [6, 7]. The USP DUB family plays roles in various cancers. USP15 regulates cell function in acute myeloid leukemia by modulating cellular redox mechanisms [8]. USP21 promotes pancreatic tumor progression and growth [9]. USP25 promotes colorectal cancer development by increasing colonic inflammation and bacterial infection [10]. USP2a, USP16, and USP22 display various important functions during PCa initiation and progression [11–13]. USP10 regulates androgen signaling and indicates poor prognosis in prostate cancer by deubiquitinating G3BP2 and H2A.Z [14, 15]We previously reported that the inhibition of specific DUBs enhanced the effects of suppressive PCa therapies [16]. Here, we explored whether other DUBs were involved in CRPC progression. We used the CRPC LNCaP-AI cell line, derived from the LNCaP cell line, to search for potential therapeutic DUB targets. A comparison of the two cell lines revealed that USP54 plays an important role in CRPC development; we discuss the implications.

## Materials and methods

### Cell lines and cell culture

The human PCa cell lines LNCaP, 22Rv1, and PC3 were purchased from the Stem Cell Bank, Chinese Academy of Sciences (Shanghai, China) and cultured in RPMI-1640 medium (L220KJ; BasalMedia, Shanghai, China) with 10% (v/v) fetal bovine serum (F7524; Sigma, St. Louis, MO, USA). The human PCa cell lines DU145 were purchased from the Stem Cell Bank, Chinese Academy of Sciences (Shanghai, China) and cultured in DMEM high glucose medium (L110KJ; BasalMedia, Shanghai, China) with 10% (v/v) fetal bovine serum (F7524; Sigma, St. Louis, MO, USA). The prostate normal cell lines BPH-1 and RWPE-1 were purchased from the Stem Cell Bank, Chinese Academy of Sciences (Shanghai, China) and cultured in Keratinocyte complete medium(C120JV; BasalMedia, Shanghai, China). The LNCaP_AI cell line was established by culturing LNCaP cells in RPMI-1640 medium with 10% (w/v) charcoal-stripped FBS (CS-FBS) for > 6 months. All cells were maintained at 37℃ with 5% (v/v) CO_2_. Culture media were changed every 2–3 days depending on cell status and density.

## Plasmids and lentivirus

Short hairpin RNA sequences targeting USP54 (shUSP54#1: 5ʹ-GTTCTGTGATTCTC AGCTTAA-3ʹ; shUSP54#2: 5ʹ-CCACAGGCAAGGTTTACCTAA-3ʹ) and a control shRNA sequence (shCON: 5ʹ-GCTCCGTGAACGGCCACGAGT-3ʹ) were cloned into the pLKO.1 vector. A lentivirus targeting USP54 was produced by co-transfection of HEK293Tcells with shRNA and psPAX2 (12,260; Addgene, Watertown, MA, USA), and pCMV-VSVG (8454; Addgene, Watertown, MA, USA) with PEI 40 K (G1802; Servicebio, Shanghai, China). The lentivirus-containing supernatants were collected after 48 and 72 h, then used to infect 22Rv1 and PC3 cells. Puromycin (5 μg/mL) (Sigma-Aldrich) was utilized to isolate stable transformants of such cells.

## Western blotting (WB)

Cells were gently washed with 3 times with phosphate-buffered saline (PBS) and dissolved in lysis buffer; Protein samples were separated by sodium dodecyl sulfate–polyacrylamide gel electrophoresis then transferred to polyvinylidene fluoride membranes. The membranes were blocked by 5%bovin serum albumin (BSA) in Tris-buffered saline with Tween (TBST) 3 times and then incubated with primary antibodies at 4℃ overnight. Subsequently, the membranes were hybridized with secondary antibody that correspond to primary antibody at room temperature for 1 h and washed in TBST 3 times. The signal density was visualized on Tanon Imaging System (Tanon-5200). Antibodies used in WB assay were as follow: β-actin (GPSG190114AA; GenePharma, Shanghai, China), AR(D6F11; CST, Danvers, MA, USA), PSA(10,679–1-AP; Proteintech, Wuhan, China), USP54(HPA063665; Sigma, St. Louis, MO, USA), Vinculin(66,305–1-Ig; Proteintech, Wuhan, China). All antibodies were diluted to the recommended concentration according to the manufacturer’s instructions.

## Real-time polymerase chain reaction (PCR)

Total RNA was isolated using Total RNA Extractor (TRIzol) (B511311-0100; Sangon Biotech, Shanghai, China), in accordance with the manufacturer’s instructions, then converted to cDNA using the ABScript III RT Master Mix for qPCR with the gDNA Remover (RK20429; ABclonal, Wuhan, China). Quantitative real-time polymerase chain reaction (qRT-PCR) was performed with QuantStudioTM Real-Time PCR Software v1.7.1 (Applied Biosystems; USA) and the 2X Universal SYBR Green Fast qPCR Mix (RK21203; ABclonal, Wuhan, China). The 2^−ΔΔCt^ method was used to analyze the relative levels of USP54 DNA expression; GAPDH served as the internal control. The primers were: USP54 forward: 5ʹ-GATGTTTGCACCTCGAAGCTC-3ʹ; USP54 reverse: 5ʹ-TCCCATGCACTTGTGAGTTGT-3ʹ; GAPDH forward: 5ʹ-TCCTGTTCGACAGTCAGCCGCA-3ʹ; GAPDH reverse: 5ʹ-ACCAGGCGCCCAAT ACGACCA-3ʹ; KLK3 forward: 5ʹ-CACAGGCCAGGTATTTCAGGT-3ʹ; KLK3 reverse: 5ʹ-GAGGCTCATATCGTAGAGCGG-3ʹ; UBE2C forward: 5ʹ-AGTGGCTAC.

CCTTACAATGCG-3ʹ; and UBE2C reverse: 5ʹ-TTACCCTGGGTGTCCACGTT-3ʹ.

## Cell viability and colony formation assays

Cell growth evaluation was conducted with the cell counting kit-8 assay system (SB/CCK-8; Share-bio, Shanghai, China). Cells (1,000 per well) were seeded into 96-well plates at 100 μL/well, and the medium was replaced with medium containing 10% (w/v) CCK-8 solution at the indicated times. Absorbance at 450 nm was measured after 3 h of further incubation at 37℃ with 5% (v/v) CO_2_. Colony formation was assayed by culturing cells in 6-well plates (1,000 cells per well) for > 12 days until colonies were visible; the cells were then washed twice with phosphate-buffered saline, fixed with methanol for 10 min, and stained with 0.5% (w/v) crystal violet at room temperature. The colonies were photographed and cell numbers were counted.

## Animal experiments

PC3 cells (5 × 10^5^) infected with lentiviruses targeting USP54 or a control gene were mixed with Matrigel (1:1 v/v) (356,234; Corning, Inc., Corning, NY, USA) and subcutaneously injected into BALB/c nude mice (SipeiFubiotech, Beijing, China). A tumor-free xenograft was defined as a xenograft that did not reach the flank. All mice were sacrificed after 35 days; the xenografts were extracted, weighed, and photographed. The experimental protocol was approved by the Experimental Animal Ethics Committee of the Department of Laboratory Animal Science, Fudan University. All the animal experiment designs are in accord with the 3R principles (Replacement, Reduction, Refinement). The experiments were approved by the Experimental Animal Ethics Committee of the Department of Laboratory Animal Science, Fudan University and the care of animals was in accordance with institutional guidelines. The BALB/c nude mice were purchased from commercial company (SipeiFubiotech, Beijing, China), which are specific pathogen free animals. All mice were housed in a monitored environment at 23 ± 1 ℃ and 50–60% relative humidity with a 12 h light/12 h dark cycles and were offered water and food ad libitum. The mice were randomly divided into two groups, and then the different cells were subcutaneously injected into the flank. The tunnel handling was used to pick a mouse up, and the mouse was restrained by three fingers. Euthanasia of mice was asphyxiation using carbon dioxide (CO_2_) followed by cervical dislocation. Using a non-precharged chamber, CO_2_ is dispensed from a commercial cylinder with fixed pressure regulator and inline restrictor controlling gas flow within 30%-70% of the chamber volume per minute to comply with 2020 AVMA Guidelines. CO_2_ flow will be maintained for > 60 s following respiratory arrest (which may take up to 5 min), followed by cervical dislocation to assure euthanasia.

## Patient gene profiles and clinical data

Total RNAs from LNCaP and LNCaP-AI cells were subjected to RNA sequencing (RNA-Seq) by Majorbio Biopham Technology (Shanghai, China). The expression profiles were analyzed on the Majorbio Cloud Platform and the data have been uploaded to Sequence Read Archive (SRA) data of NCBI (PRJNA1040316). Gene expression datasets of human PCa samples (GSE29079 and GSE35988) were respectively downloaded from the Cancer Genome Atlas (TCGA) database (http://protal.gdc.can.gov/) and the Gene Expression Omnibus (GEO) database (http://www.ncbi.nlm.nih.gov/gds/). The MSKCC and SU2C 2019 datasets were accessed via cBioPortal (http://www.cbioportal.org/). Gene set enrichment analysis (GSEA) was performed with GSEA software (version 4.2.2). The hallmark gene sets were obtained from the Broad Institute (http://www.broadinstitute.org/gsea/index.jsp).

## Statistical analysis

All statistical analyses were performed with GraphPad Prism software (version 9.0). Quantitative data are presented as means ± standard deviations (SDs). Student’s t-test and Spearman correlation analysis were utilized as appropriate. The Kaplan–Meier method was used to analyze survival data. *P*-values < 0.05 were considered statistically significant (*P < 0.05, **P < 0.01, ***P < 0.001).

## Results

### USP54 is upregulated during HSPC progression to CRPC

**Fig. 1:**
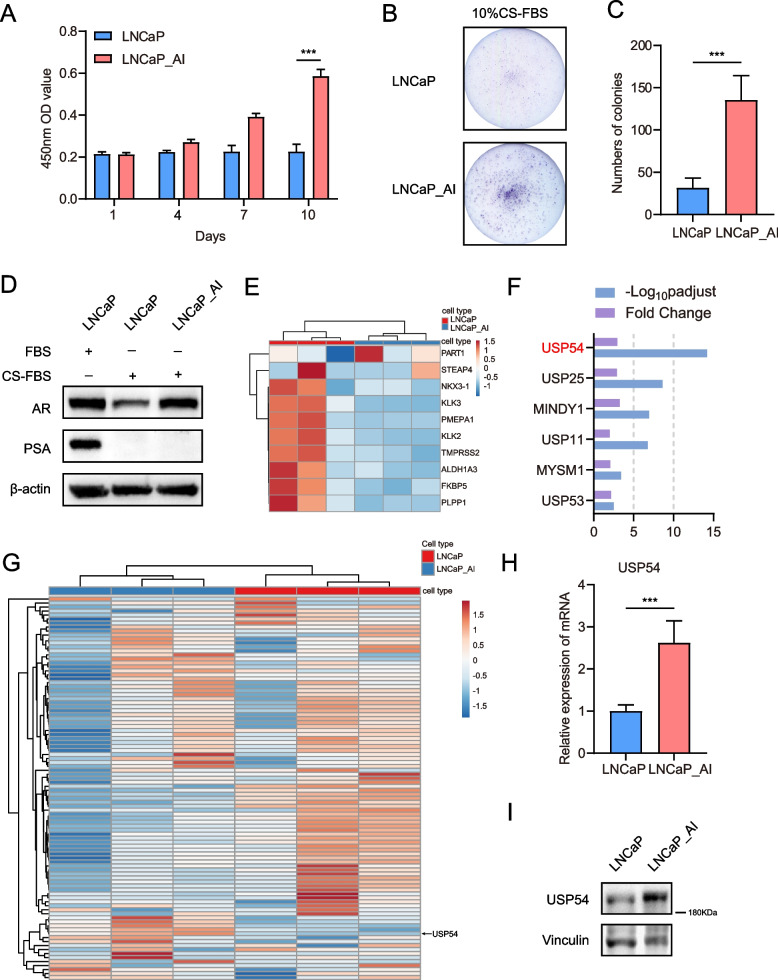
USP54 is a possible therapeutic target in CRPC cells. **A** The viabilities of LNCaP and LNCaP-AI cells cultured in 10% (v/v) CS-FBS-supplemented medium were measured using the CCK-8 assay at the indicated times. **B**, **C** Colony formation by LNCaP and LNCaP-AI cells cultured in the same medium; the colony numbers are shown. **D** Expression of indicated protein in LNCaP and LNCaP_AI by Western blotting; β-actin was used as reference gene. **E** Expression of AR-response genes by LNCaP and LNCaP-AI cells; the heat map indicates differential expression levels. **G** Expression of USP proteins by LNCaP and LNCaP-AI cells; the heat map indicates differential expression levels. **F** Upregulated DUBs, as revealed by fold changes and –log_10_ p_adjust_ values. **H** The levels of mRNA encoding USP54, as measured by qPCR (t-test; error bars represent SDs; ***P < 0.001). **I** Expression of indicated protein in LNCaP and LNCaP_AI by Western blotting; vinculin was used as reference gene

We mimicked progression from HSPC to CRPC by using a long-term androgen-deprivation culture system to establish the androgen-independent CRPC cell line LNCaP-AI from the androgen-dependent PCa cell line LNCaP. The CCK-8 assay showed that LNCaP cell proliferation in androgen-deprivation medium was significantly worse than LNCaP-AI proliferation in that medium (Fig. 1A). The colony formation capacity of LNCaP-AI cells was unaffected by androgen deprivation, in contrast to the capacity of LNCaP cells (Fig. 1B and C). Western blotting has been utilized to detect the long-term effects of AR suppression in LNCaP AI cells (Fig. 1D). Next, LNCaP and LNCaP-AI cell lines were subjected to RNA-Seq, and the expression profiles were examined by differential analysis. The AR-responsive genes detected by RNA-seq was shown in Fig. 1 E. We focused on the DUB family, which plays important roles in PCa occurrence and progression. We filtered USP expression levels in the LNCaP and LNCaP-AI cell lines (Fig. 1G). The greatest difference between cell lines was the extent of USP54 upregulation in LNCaP-AI cells (Fig. 1F). Next, we examined the levels of mRNA encoding USP54; we found that they were higher in LNCaP-AI cells than in LNCaP cells (Fig. 1H). Then, the protein expression also has been confirmed (Fig. 1I).

## USP54 expression is upregulated in PCa

**Fig. 2:**
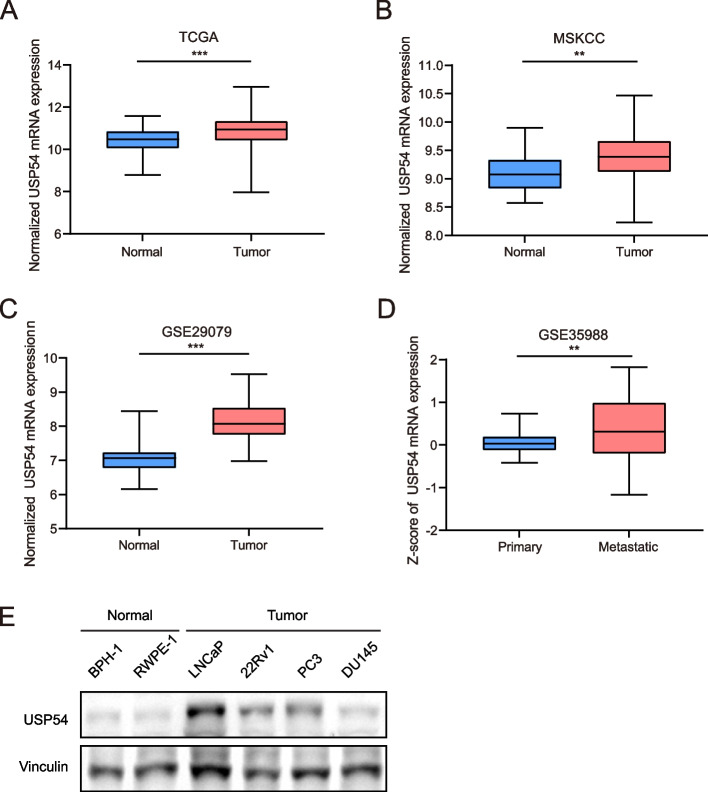
USP54 expression is upregulated in PCa tissues. **A**, **B**, **C** The expression levels of USP54 in normal prostate tissues and primary PCa tissues, as revealed by the Cancer Genome Atlas (TCGA), the Memorial Sloan Kettering Cancer Center (MSKCC) dataset, and the Gene Expression Omnibus (GEO) dataset GSE29079. **D** The expression levels of USP54 in primary and metastatic prostate cancer tissues, as revealed by the GSE35988 dataset. (t-test; *P < 0.05; **P < 0.01; ***P < 0.001). **E** Expression of indicated protein in prostate normal and tumor cells by Western blotting; vinculin was used as reference gene

We extracted USP54 expression levels from clinical PCa samples contained in the TCGA, MSKCC, and GEO databases. Compared with normal tissue, the level of mRNA encoding USP54 was upregulated in PCa tissues (Fig. 2A-C). In another GEO dataset, USP54 expression was higher in advanced PCa (metastatic tumors) than in primary PCa (Fig. 2D). In PCa cells, USP54 expression levels were abnormally elevated compared to normal prostate cells, as depicted in Fig. 2E. This elevation in USP54 expression, observed in PCa cells, indicates its potential involvement in PCa development.

## USP54 knockdown decreases CRPC cell growth in vitro

**Fig. 3:**
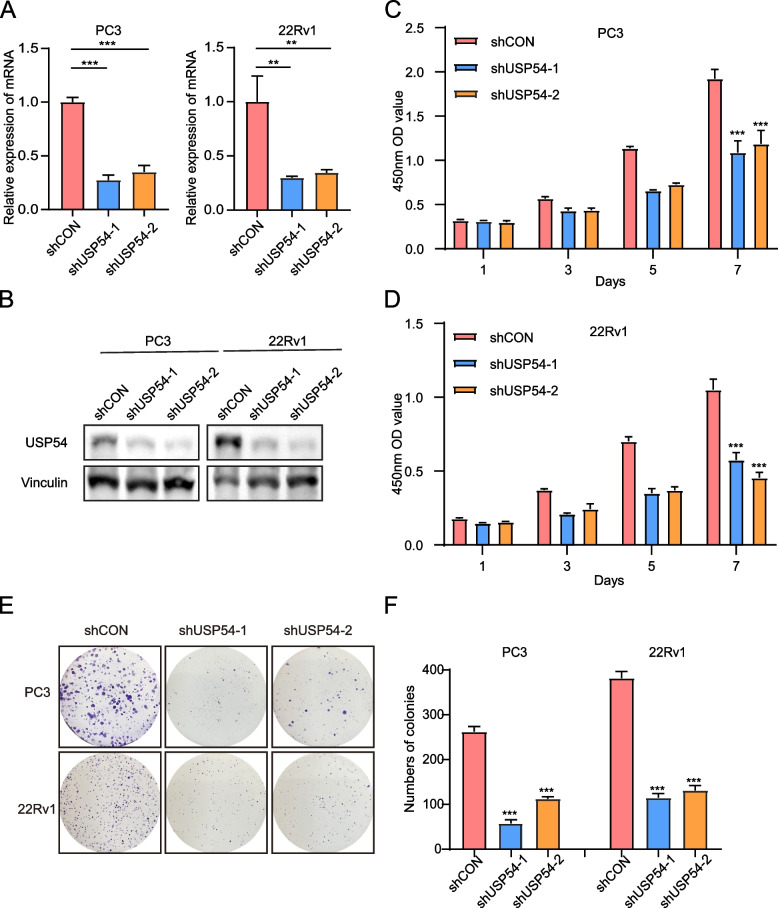
USP54 knockdown impairs CRPC cell proliferation in vitro. **A** qPCR confirmed that the shRNA provided efficient knockdown. **B** WB confirmed that he shRNA provided efficient knockdown. **C**, **D** The viabilities of PC3 and 22Rv1 cells expressing shRNAs that target a control sequence or USP54, measured using the CCK-8 assay at the indicated times. **E**, **F** Colony formation assays of PC3 and 22Rv1 cells transfected with the indicated shRNAs; the colony numbers are shown. (t-test; error bars represent SDs; **P < 0.01; ***P < 0.001)

To investigate the biological function of USP54 in CRPC cells, we silenced USP54 in PC3 and 22Rv1 cell lines (CRPC lines); RT-PCR and WB confirmed efficient USP54 knockdown (Figs. 3A and B). USP54 knockdown substantially reduced CRPC cell growth, as revealed by the CCK-8 assay (Figs. 3C and D); it also significantly reduced cell colony sizes and cell numbers compared with those characteristics in the control group (Fig. 3E). The colony numbers are shown in Fig. 3F. Collectively, the data suggested that USP54 targeting inhibited CRPC cell proliferation in vitro.

## USP54 downregulation influences CRPC cell growth in vivo

**Fig. 4:**
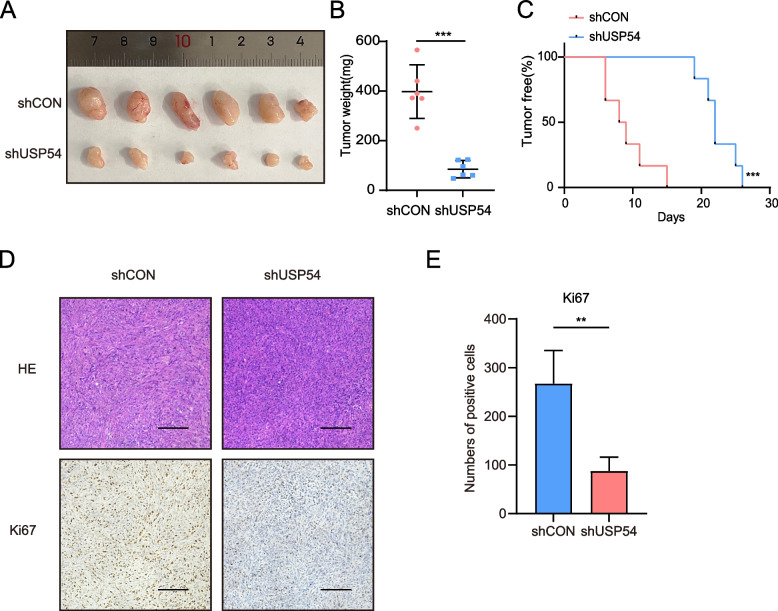
USP54 downregulated impedes CRPC cell growth in vivo. **A** PC3 cells (5 × 10^5^) expressing shRNA targeting a control sequence and USP54 were suspended in Matrigel (1:1 v/v) and subcutaneously implanted into 6-week-old nude mice (*n* = 6); the mice were sacrificed after 35 days, and the volumes of xenograft tumors were determined. **B** The weights of xenografts in each group. Error bars: means ± SDs (Mann–Whitney test; *n* = 6; ***P < 0.001). **C** Kaplan–Meier analysis of tumor onset times (log-rank test; ***P < 0.001). **D** Hematoxylin–eosin (HE) and Ki-67 immunohistochemical staining of tumor xenografts (scale bar: 100 μm). **E** The numbers of cells expressing Ki-67 (Mann–Whitney test; **P < 0.01)

To confirm the effect of USP54 on CRPC cell growth in vivo, PC3 cells stably expressing shUSP54 and shCON were subcutaneously injected into 6-week-old nude mice (*n* = 6); after 35 days, the xenografts were removed. Xenografts were larger and significantly heavier in the control group than in the USP54-knockdown (test) group (Fig. 4A and B). USP54 inhibition delayed tumorigenesis (Fig. 4C). Xenograft immunohistochemical staining revealed that the level of the proliferation marker Ki-67 was lower in the USP54-knockdown group (Fig. 4D and E). Thus, the loss of USP54 significantly reduced CRPC cell proliferation in vivo.

## USP54 affects androgen receptor (AR) signaling in PCa

**Fig. 5:**
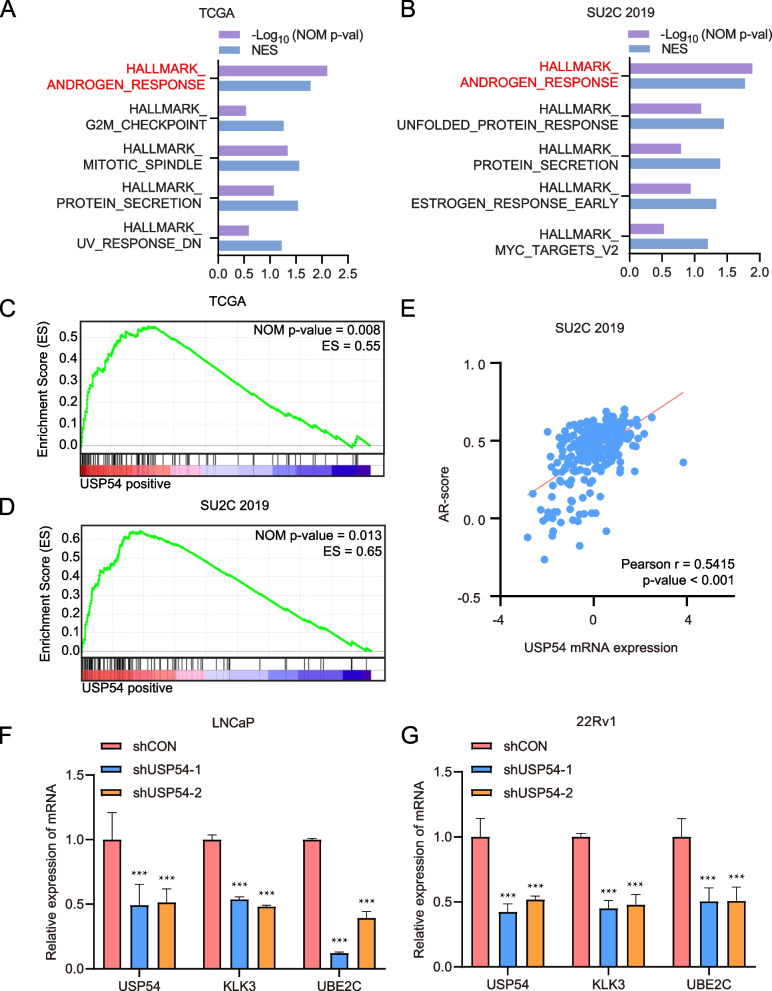
USP54 affects AR signaling in PCa. **A**, **B** GSEA of hallmark gene sets indicated that the androgen-response set was the most highly enriched in patients exhibiting high-level USP54 expression in the indicated PCa cohorts. The results are ranked according to normalized enrichment score and *P*-value. **C**, **D** The androgen-response gene set was used for GSEA of the various datasets based on USP54 transcriptional level. **E** The SU2C 2019 dataset was analyzed in terms of the correlation between the level of mRNA encoding USP54 and AR signaling activity. (F-G) Bar graph showing the levels of mRNAs encoding representative AR downstream target proteins after USP54 inhibition (t-test; ***P < 0.001)

To investigate mechanisms by which USP54 inhibition could reduce CRPC cell growth, we subjected the TCGA and SU2C 2019 datasets to GSEA (Fig. 5A and B). The ranked, relative, normalized enrichment scores and *P*-values revealed that the androgen-response gene set was the most highly enriched set in patients expressing high levels of USP54 (Fig. 5A-D). In the SU2C 2019 CRPC cohorts, we found a strong positive correlation between the USP54 expression level and AR signaling activity (Fig. 5E). Next, we measured the levels of mRNAs encoding classical AR signaling downstream target genes after USP54 knockdown in both LNCaP and 22Rv1 cells. The expression levels of both the androgen-dependent KLK3-encoding gene and the androgen-independent UBE2C-encoding gene [15] decreased after USP54 inhibition (Fig. 5F and G). Thus, USP54 may affect CRPC AR signaling activity, which influences tumor growth.

## Discussion

ADT (surgical or medical castration) remains the primary treatment for patients with metastatic PCa [1, 17]. However, tumor regression persists for a median of only 18–24 months; tumors become castration-resistant, and ADT subsequently does not inhibit growth [18]. We mimicked ADT in vitro; the LNCaP-AI cell line was subjected to long-term androgen-deprivation culture. Similar to previous reports [19, 20], we found that LNCaP-AI cells proliferated more rapidly than LNCaP cells under androgen-deprivation conditions. Thus, we used LNCaP-AI cells to identify potentially upregulated deubiquitinating enzymes in CRPC; we subjected the cells to RNA-Seq. USP54 exhibited the greatest upregulation in CRPC cells, compared with HSPC cells.

USP54 is a member of the DUB family that is involved in the progression of various tumors. USP54 is overexpressed in intestinal stem cells; USP54 downregulation in colorectal carcinoma cells impedes tumorigenesis [21]. In esophageal adenocarcinomas, the USP54-encoding gene is sometimes fused with the CAMK2G-encoding gene [22]. USP54 promotes gastric cancer progression by deubiquitinating PLK4 [23]. However, the roles of USP54 in other cancers, especially PCa, remain unclear. Here, we explored the USP54 mRNA expression levels in publicly available datasets; the levels were abnormally elevated in both early and advanced PCa tumors. USP54 gene disruption significantly inhibited CRPC cell proliferation, both in vitro and in vivo. Overall, the data suggest that USP54 potentially affects CRPC oncogenesis and progression.

PCa growth is enhanced by AR signaling. Under ADT selection pressure, AR activity may be maintained via gene amplification [24] or the expression of AR-V7 that lacks an intact ligand-binding domain for androgen-binding [25], causing antagonistic drug responses to become agonistic [26]. Constitutive AR signaling is required for most castration-resistant tumor growth [27]. However, the associated molecular changes remain poorly understood. We used GSEA to investigate potential mechanisms underlying the effects of USP54 on PCa cell growth. Bioinformatics analysis revealed that USP54 expression was positively correlated with both the androgen response and AR signaling in PCa tissues. USP54 affected androgen-dependent and -independent gene expression in PCa cells. USP54 may regulate CRPC cell proliferation by maintaining AR signaling activity. Thus, USP54 targeting might inhibit AR signaling and reduce CRPC cell growth. We hypothesize that USP54 may influence AR signaling activity in PCa cells by deubiquitinating a protein key to AR signaling activation, However, the specific substrate of USP54 in prostate cancer remains unidentified, necessitating further research. Our study reveals USP54’s impact on cell growth in both AR-positive and AR-negative PCa cells, hinting at the involvement of AR-independent pathways in CRPC development. As a deubiquitinase, USP54 governs the degradation of multiple proteins through the ubiquitin–proteasome system, potentially affecting various biological processes and growth signals.

In our research, we observed an upregulation of certain DUBs during the progression from HSPC to CRPC. USP25 and USP11, in particular, merit further investigation. USP25 is known to interact with TNKS, influencing prostate cancer cell proliferation via the WNT signaling pathway, which plays a crucial role in PCa progression [28]. Meanwhile, USP11 expression correlates with key prognostic factors like PSA levels and Gleason scores [29]. However, the specific role of USP11 in the transition from HSPC to CRPC remains to be elucidated.

Our study has several limitations. Firstly, the mechanism by which USP54 regulates AR signaling remains uncertain. Investigating whether USP54 directly interacts with AR, potentially inhibiting its ubiquitination and degradation, is crucial. Secondly, our experiments exclusively used androgen-independent cell lines. Therefore, understanding the biological function of USP54 in androgen-dependent prostate cancer cells requires further exploration.

In conclusion, our results reveal the pivotal role of USP54 during HSPC transformation into CRPC, and they indicate that USP54 targeting could significantly inhibit PCa proliferation in vivo. Additionally, USP54 affects AR signaling activity in PCa cells; this may explain why PCa modulates cell growth.

### Supplementary Information

**Additional file 1.**

## Data Availability

Sequence data that support the findings of this study have been deposited in the NCBI with the primary accession code of PRJNA1040316.
