# Supplementary figures and images for: USP54 is a potential therapeutic target in castration-resistant prostate cancer
Source: BMC Urol. 2024 Feb 6;24:32. doi: 10.1186/s12894-024-01418-7 (PMC10845770; doi:10.1186/s12894-024-01418-7)

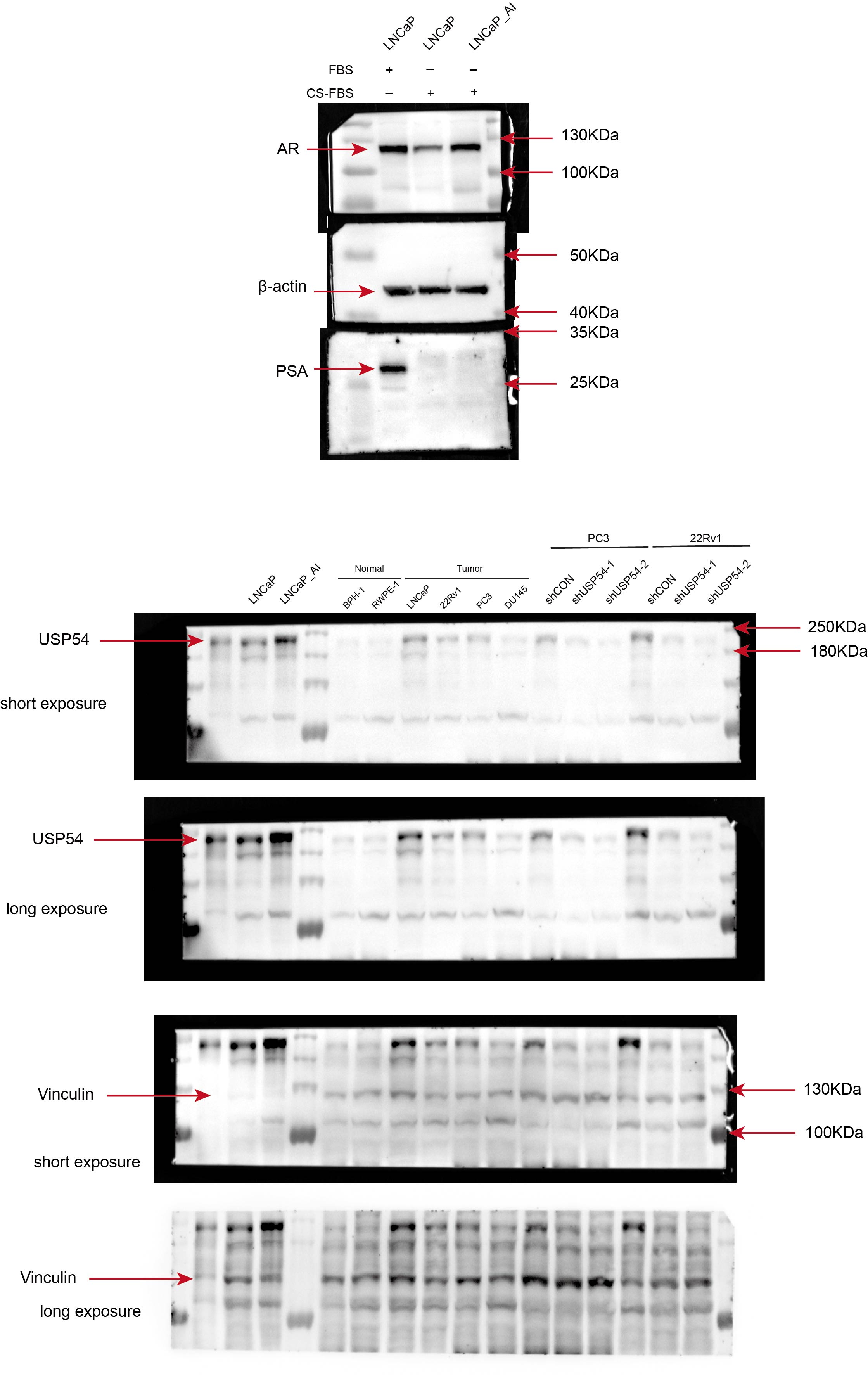

Supplement: Supplementary file 1 — Additional file 1. [file 12894_2024_1418_MOESM1_ESM.jpg]
